# Alternative Extraction and Downstream Purification Processes for Anthocyanins

**DOI:** 10.3390/molecules27020368

**Published:** 2022-01-07

**Authors:** Ana N. Nunes, Alexandra Borges, Ana A. Matias, Maria Rosário Bronze, Joana Oliveira

**Affiliations:** 1 iBET, Instituto de Biologia Experimental e Tecnológica, 2780-901 Oeiras, Portugal; annunes@ibet.pt (A.N.N.); ana.matias@cleverleaves.com (A.A.M.); mbronze@ibet.pt (M.R.B.); 2 ITQB, Instituto de Tecnologia Química e Biológica António Xavier, Universidade Nova de Lisboa, 2780-157 Oeiras, Portugal; 3 Laboratório Associado para a Química Verde—REQUIMTE, Departamento de Química e Bioquímica, Faculdade de Ciências da Universidade do Porto, Rua do Campo Alegre S/N, 4169-007 Porto, Portugal; alexandraborgespnf@gmail.com; 4 iMed.Ulisboa, Instituto de Investigação do Medicamento, Faculdade de Farmácia da Universidade de Lisboa, Avenida das Forças Armadas, 1649-019 Lisboa, Portugal

**Keywords:** anthocyanins, downstream processes, alternative solvents, counter-current chromatography

## Abstract

Anthocyanins are natural pigments displaying different attractive colors ranging from red, violet, to blue. These pigments present health benefits that increased their use in food, nutraceuticals, and the cosmetic industry. However, anthocyanins are mainly extracted through conventional methods that are time-consuming and involve the use of organic solvents. Moreover, the chemical diversity of the obtained complex extracts make the downstream purification step challenging. Therefore, the growing demand of these high-value pigments has stimulated the interest in designing new, safe, cost-effective, and tunable strategies for their extraction and purification. The current review focuses on the potential application of compressed fluid-based (such as subcritical and supercritical fluid extraction and pressurized liquid extraction) and deep eutectic solvents-based extraction methods for the recovery of anthocyanins. In addition, an updated review of the application of counter-current chromatography for anthocyanins purification is provided as a faster and cost-effective alternative to preparative-scale HPLC.

## 1. Introduction

Anthocyanins are a well-known class of phenolic compounds responsible for the red, violet, and blue colors displayed by several flowers, vegetables, fruits, and fruit-derived products, such as fruit juices and red wines [1,2]. Over the years, these pigments have been applied as natural colorants in food and cosmetics industries to replace synthetic dyes [3,4]. Along with their vibrant colors, anthocyanins are also known by their biological properties, including their antioxidant activity [5,6], anti-cytotoxic, and anti-carcinogenic effects in different cell culture systems [7,8,9,10,11]. Therefore, anthocyanins are not only used as colorants to improve the overall food and cosmetics appearance, but also to enhance their health promoting properties. The Joint FAO/WHO Expert Committee on Food Additives claimed that anthocyanin-containing extracts had a very low toxicity and no adverse impacts on health after their oral consumption [12]. This is important since functional foods are being rapidly integrated into the market and increasingly accepted by consumers. 

Considering the general properties of anthocyanins, their extraction from natural sources have been increasingly explored [3,13,14,15]. The most common techniques for anthocyanins extraction include the use of organic solvents [16,17], subcritical water [18], supercritical CO_2_ [19], pressurized liquid [20], microwave, and ultrasound extractions [21,22]. More recently, the use of neoteric solvents that comprise low-temperature molten salts (ionic liquids and eutectic solvents) and supercritical fluids has been described as a greener alternative to traditional organic solvents for the extraction of these pigments [23]. 

However, there are drawbacks associated with some of these techniques, namely the high costs of processes, the low extraction yields, and the use of solvents that are harmful for the environment. This increases the need for the development of new methods to extract anthocyanins at a high yield and a low cost. Bearing this in mind, this review describes the use of neoteric solvents (deep eutectic solvents and compressed fluids) as alternative methods for the extraction of anthocyanins that have been reported in the last few years. The application of counter-current chromatography for anthocyanins purification is also described.

## 2. Alternative Solvents

Amongst all extraction techniques, solvent extraction remains the most significant methodology widely used for the extraction of anthocyanins from different food sources. With this technique, there is the possibility to operate in a continuous mode. The required equipment is usually simple and it is possible to select an efficient solvent for their extraction based on its solubility, cost and safety. Hence, polar solvents including ethanol, methanol, and acetone, are the most common solvents applied in the extraction of phytochemicals due to their similar polarity [24]. However, the toxicity of most organic solvents is a major disadvantage and research has been performed to overcome this by using low toxicity, easy to recycle, or inert alternative solvents.

Ionic Liquids (IL) are molten salts that melt at temperatures below 100 °C and are composed mainly by one type of cation or anion. Their characteristics allow their application in biocatalytic processes and extraction techniques [25,26,27]. These solvents gained particular recognition as substitutes of volatile organic solvents as the importance of the Green Chemistry field started to grow [28]. ILs can be expensive and cannot be generally classified as “green” due to their non-degradability, which is an important feature for their use in sustainable extraction processes. Therefore, a new category of solvents, known as deep eutectic solvents (DES), was developed comprising both the unique physicochemical characteristics of ILs and the advantage of being biodegradable and non-toxic materials [29]. 

### 2.1. Deep Eutectic Solvents

DES are a category of green solvents that are often associated to ILs due to common characteristics, namely high thermal stability, low volatility and low vapor pressure [30]. Natural deep eutectic solvents (NADES) are particularly composed of natural compounds produced by cell metabolism and they present similar properties to DES [31]. DES are mainly formed by two components, one usually being a hydrogen bond acceptor (HBA) (e.g., quaternary ammonium, tetraalkylammonium or phosphonium salts) and a hydrogen bond donor (HBD) (acids, alcohols, amines or carbohydrates) [31,32]. The charge delocalization that occurs through hydrogen bonding between the HBA and the HBD is responsible for an important decrease in the melting point of the mixture, in comparison to the melting points of the individual components [33,34]. In contrast to ILs, DES are less expensive to produce, have a higher biodegradability, and lower toxicity, which makes them more attractive in their application in new sustainable extraction options [35]. 

DES can be generally classified as Type I when the combination consists of a quaternary ammonium salt and a metal chloride, Type II when it combines a quaternary ammonium salt and a metal chloride hydrate, Type III when it consists of a quaternary ammonium salt and a HBD (typically an amide, carboxylic acid or polyol), and Type IV that consists of the combination of a metal chloride hydrate and HBD [30,36]. 

More recently, another kind of DES has also been introduced, Type V, which is composed only by nonionic molecular HBAs and HBDs [37]. Over the last years, ternary DES, prepared by the mixture of one type of HBA and two types of HBD, have also been an important research topic that have already been applied in different areas such as carbon dioxide absorption [38], extraction of lignin [39], and reversible absorption of NH_3_ [40]. These ternary systems have particularly a lower viscosity and melting point than binary DES, although their physicochemical characteristics are still under study [41,42,43].

The preparation of these solvents can be performed by: (i) heating a mixture of each component until a homogeneous liquid is obtained, or (ii) dissolving the components in distilled water and lyophilizing until a constant weight is reached [27,44]. Hence, when applied in extraction methods, these solvents represent a key advantage due to their simple preparation. In an extraction process, a sample is added to the solvent and the mixture can be stirred at a fixed temperature for a given amount of time [45]. Auxiliary extraction techniques, mainly microwave-assisted and ultrasound-assisted extractions, are commonly applied during extraction with DES [31,46]. Ultrasound-assisted extraction is shown to promote extraction by increasing the process yield and reducing the extraction time. On the other hand, microwave-assisted extraction reduces energy consumption, which is in accordance with the principles of “green” extraction [47,48,49]. 

### 2.2. Anthocyanins Extraction Using DES

The use of DES as extraction solvents for phenolic compounds has been widely studied. Their high ability to extract these molecules is mainly due to H-bond interactions that take place between the solvent and the phenolic compounds [27,50]. The extraction of anthocyanins from different natural sources using DES has been reported by several authors. Table 1 shows some examples of the different combinations of DES used for the extraction of these compounds. It is important to note that the anthocyanin recovery differs not only based on the combination of DES used but also the auxiliary extraction techniques used, such as ultrasound or microwave assisted extraction. Anthocyanin extraction using DES usually starts with the screening of several DES/NADES that are prepared, varying the components, HBA:HBD ratio and water percentage. As a control experiment, extraction using the conventional solvents (aqueous solutions of ethanol or methanol) is done. In these studies, at least one HBA:HBD combination presents higher extraction yields than the conventional solvents. In addition, as it is evidently shown in Table 1, choline chloride is the HBA mainly used not only due to its properties, but also due to its low price.

Bubalo et al. used a mixture of choline chlorine: oxalic acid (1:1) with 25% water and this solvent showed 5- and 2-fold higher extraction yields of total anthocyanins than water and aqueous methanol solutions, respectively [52]. Similarly, Zannou et al. observed that the extraction of anthocyanins performed with sodium acetate:formic acid (1:2) leads to extraction yields of 1.03-, 1.09-, and 1.23-fold higher than 70% ethanol, distilled water, and 80% methanol, respectively [65]. Among the different physicochemical properties of DES, polarity represents the most important one and a wide range of polarity can be usually achieved according to the mixture composition. DES prepared from carbohydrates present higher polarities than those composed of short chain alcohols and some polar aprotic solvents [29,66]. In general, higher extractions yields of anthocyanins are obtained when organic acids, such as lactic and oxalic acids, are used as HBD [52,55,56]. This can be explained by two main factors: the polarity and the acidity character of the final mixture. As the functional groups typically associated with DES/NADES are hydroxyl, carboxyl, and amine groups, hydrogen-bonding interactions are formed between the solvent molecules and the hydroxyl groups of anthocyanins [50,67]. On the other hand, the acidic medium also contributes for a high extraction yield of these pigments, since the flavylium cation form of these molecules is stabilized in those conditions which improves both extraction and stability of anthocyanins. Bosiljkov et al. used different NADES to extract anthocyanins from wine lees and observed that higher extraction yields were obtained with more acidic NADES (pH < 3), namely when using malic and citric acids as HBD, with choline chloride as a HBA [47]. Moreover, it is common to observe that the color intensity of DES/NADES-anthocyanin extracts is higher at lower pH values [67]. Another important parameter that influences extraction is the viscosity of the solvents that is usually modulated by altering the water percentage. In all studies, it was reported that lower values of apparent viscosity yielded to higher anthocyanins extraction but also facilitates the use of auxiliary techniques, with ideal values of water percentage around 20%.

A major advantage of using DES is that the compound selectivity can be manipulated using these solvents. For instance, different fruits or plants present a different anthocyanin composition, and changing HBA/HBD composition and ratio allows to obtain higher extraction yields of a given target molecule. Alrugaibah et al. used several eutectic mixtures to extract anthocyanins from cranberry pomace and concluded that different compositions showed dissimilar selectivity towards each anthocyanin and procyanidin [53]. The same authors observed that a solvent prepared with a mixture of citric acid:maltose (4:1) and 35.5% water, selectively extracted a higher amount of cyanidin-3-*O*-arabinoside and peonidin-3-galactoside in comparison to other anthocyanins, including cyanidin-3-galactoside, cyanidin-3-*O*-glucoside, peonidin-3-*O*-glucoside, and peonidin-3-*O*-arabinoside. Moreover, the highest extraction value of total anthocyanins (1.54 ± 0.05 mg/g) was obtained using a mixture of choline chloride:lactic acid (1:5) with 20% of water, and three of the prepared DES extracted approximately 1.6- fold more anthocyanins than 75% ethanol [53].

These solvents can represent an innovative and promising way to obtain anthocyanins from natural sources. In fact, several studies have already demonstrated the capacity of eutectic mixtures to act not only as an extraction solvent for anthocyanins, but also to play an important role on their red color stabilization and on the enhancement of their bioactivity. Velásquez and co-workers used *L. chequen* (Molina) A. Gray fruit and were able to create edible films using anthocyanin-rich extracts obtained with choline chlorine:glycerol (4:6), glycerol:glucose (8:1) and tartaric acid:glycerol (1:4). In addition to glycerol often being used in the preparation of NADES, polyalcohols also show improved extraction of bioactive compounds and have applications in many fields such as cosmetic and food industries [58,68]. Moreover, the prepared NADES anthocyanin-rich extracts showed a higher antimicrobial activity than that of the ethanolic extracts, which indicates that these materials can improve the antibacterial properties of packaging materials [58]. In another research, Grillo et al. investigated the antiproliferative activity of a blueberry peel extract in NADES. The MTS (3-(4,5-dimethylthiazol-2-yl)-5-(3-carboxymethoxyphenyl)-2-(4-sulfophenyl)-2H-tetrazolium) assay was performed in HeLa and HaCat cell lines and the results confirmed that NADES prepared with choline chlorine:lactic acid (1:1) can improve the biological activity of extracts [51]. 

A common problem found during the use of anthocyanins is their degradability during storage. Zannou et al. showed that using a mixture of sodium acetate:formic acid (1:2) as solvent, anthocyanins were less prone to degradation during eighteen days of storage at −20 °C, 4 °C and 20 °C, when compared to their degradation in water. This emphasizes once again the importance of the strength of intramolecular and intermolecular interactions between the solvents and these pigments [65]. More recently, Souza et al. studied the stability of cyanidin-3-*O*-glucoside, extracted from blackberries, dissolved in different binary and ternary DES. During color evaluation, a red color intensity was observed for eutectic mixtures composed by choline chlorine:ethylene glycol (1:2), choline chlorine: glycerol (1:2), and choline chlorine: glycerol:ethylene glycol (1:1:1) which is related with the stabilization of the flavylium cation form of cyanidin-3-*O*-glucoside. Authors also concluded that the stability during storage of cyanidin-3-*O*-glucoside was higher when dissolved in glycerol-based DES [69]. Thus, DES represent a promising alternative for anthocyanin extraction, displaying not only a great capacity for anthocyanin extraction, but also the possibility of enhancing their chemical and biological properties, which allows DES to stand out from other conventional and more harmful solvents. 

## 3. Compressed Fluid-Based Extraction Techniques 

### 3.1. Supercritical Carbon Dioxide Extraction

Supercritical fluid extraction (SFE) is an environmentally friendly separation process based on the use of supercritical fluids as an alternative to organic solvents to extract target components from a solid matrix [70,71]. A supercritical fluid is defined as a substance above its critical temperature and pressure, exhibiting low viscosity and relatively high diffusivity, which provide appreciable penetrating power in the solid matrix with a rapid mass transfer rate [72,73,74,75,76]. Moreover, supercritical fluids have a density close to liquids that can be easily modified by tuning the pressure and the temperature [72,74,75,76]. Generally, the targeted compound solubility increases with the increase of the extraction pressure, which is directly a result of the increased solvent density [76,77]. The effect of extraction temperature is more complex and at constant pressure, an increase in temperature increases the targeted compound solubility in supercritical fluids due to it vapor pressure enhancement. On the other hand, an increase in temperature also decreases the solvent density, reducing the solubility of the targeted compound in the supercritical fluid. This results in a phenomenon called the “crossover effect” where the targeted compound solubility is no longer dependent on the supercritical fluid density [77]. Therefore, these properties enable to fine-tune the solvent extracting power and selectivity of a supercritical fluid toward a target compound.

Although several fluids have been used for SFE, carbon dioxide (CO_2_) is by far most attractive and has been most widely used in food applications. CO_2_ present several advantages including mild critical conditions (31.1 °C and 73.8 bar) that are easily attainable, non-toxic, non-inflammable, non-explosive, relatively inert to several media, inexpensive and readily available in pure form [72,74,75,76,77,78,79]. Due to these characteristics, the European Food Safety Authority and the United States Food and Drug Administration have assigned CO_2_ as a generally recognized as safe solvent. In addition, the extraction processes using supercritical CO_2_ preserve the bioactivity of the compounds due to the absence of light and oxygen in the system and low operating temperatures [80]. Moreover, the separation of compounds using supercritical CO_2_ is clean with little-to-no residue as the solvent is gaseous at room temperature and pressure [74,75,77]. Furthermore, since CO_2_ gas stream can be recycled, supercritical extraction using CO_2_ can be regarded as an environmentally-friendly process [73,76]. 

However, supercritical CO_2_ is not suitable for the extraction of polar compounds due to its low polarity [77]. Hence, the addition of a modifier can significantly improve the extraction efficiency of polar substances. These polar co-solvents are added in small amounts to induce substantial changes of the solvent properties of pure supercritical CO_2_ [73]. The target compound solubility in the supercritical CO_2_ is enhanced as a consequence of positive hydrogen bonding interactions between co-solvent and polar solutes in the supercritical phase [72,75]. Within organic solvents, ethanol is the most environmentally favorable and healthy, being accepted as a “biosolvent” for food applications [79]. The only limitations for the direct industrial application of SFE is the high investment cost and more qualified manpower required. For this reason, SFE should be only considered for high added value products or those required compliance with strict environmental regulations [72,80].

**Figure 1:**
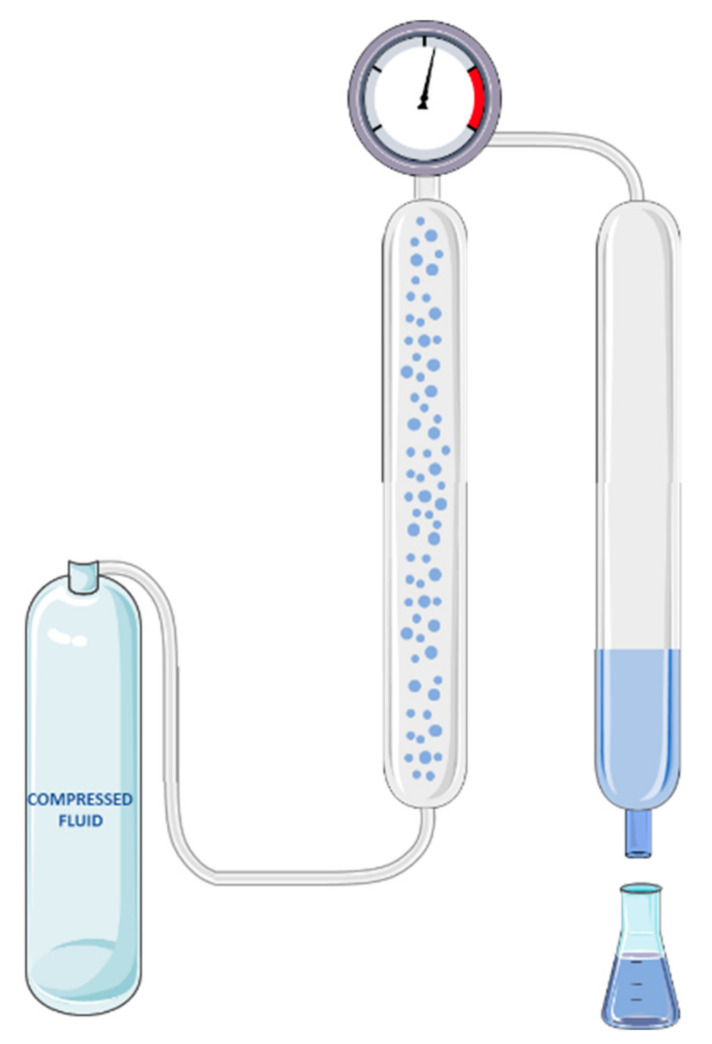
Schematic illustration of the compressed fluid-based extraction process. This figure was made with some free images available on the Smart Servier Medical Art website (https://smart.servier.com, last accessed on 22 December 2021).

SFE process is divided in two main steps (Figure 1): (1) dissolution of target compounds that are present on the solid matrix by the supercritical fluid and (2) separation of the extracted compounds from the supercritical fluid after the expansion [73,81]. During SFE, the supercritical fluid is pressurized and heated through a solvent pump and a heat exchange before entering the extractor. The desired extraction conditions are maintained by the operation of a back pressure regulating valve and temperature controllers attached to the extraction vessel [82]. The supercritical solvent is absorbed by the raw material, promoting the dilatation of the matrix structures, increasing the extraction rate. Simultaneously, the soluble compounds are dissolved by the supercritical solvent. Afterwards the separation of the solute from the supercritical fluid is achieved in the separator by pressure reduction and/or temperature increase, yielding a solvent-free extract. The extract is consequently collected at the separator and the gas can be recycled or released to atmosphere [81].

SFE represents an interesting alternative technique to conventional extraction for industrial application. The first successful industrial application was the decaffeination of coffee beans using supercritical CO_2_, patented in 1964. Currently, the use of supercritical fluids is widely applied in different industrial processes including the extraction of flavor, fragrance, essential oils, algae, biodiesel.

### 3.2. Pressurized Liquid Extraction

Pressurized liquid extraction (PLE), also known as accelerated solvent extraction in the analytical extraction area, pressurized fluid extraction, pressurized solvent extraction or enhanced solvent extraction, is an alternative solid–liquid extraction process based on the use of organic solvents at elevated temperature and pressure (always below their critical point) for the recovery of bioactive compounds from natural matrices [71,83,84]. Subcritical extractions are carried out under high pressure to maintain the liquid state of solvent, allowing extraction temperatures above its boiling point [85].

Elevating the temperatures of solvents above their atmospheric boiling points generally promote higher target compound solubility by increasing both diffusion and mass transfer rate between the matrix and the solvent, coupled with the solvent ability to disrupt the target compound-matrix interactions [85]. Moreover, high temperatures reduce the viscosity and surface tension of the solvent, and facilitate the extraction rate. On the other hand, high pressure forces solvent into matrix pores, allowing better contact between the solvent and the targeted compounds [85,86,87]. Therefore, the combined use of high pressures and temperatures provides a faster extraction process, reduced solvent consumption, and high extraction efficiencies with improved recovery rates compared to conventional techniques [83,85,88].

The solvent selection is based on the solubility characteristics of the target compound. Pressurized solvents present a great flexibility due to the solvent physical–chemical properties, namely the viscosity, diffusivity, density, and dielectric constant. These properties can be tailored by adjusting the temperature and the pressure of the system [88]. The solvents most commonly applied are water, ethanol, and their mixtures. Consequently, PLE shown to be effective when extracting both polar and moderately non-polar compounds from different matrices [84,89]. 

During PLE process, the sample is placed into the extraction vessel and the solvent is pressurized and heated until the desired combination of pressure and temperature variables are reached [82]. The extraction process can be accomplished in two modes: static or dynamic. In the static mode, after the extraction time, the system is rinsed with fresh solvent in order to transfer the extract to the collection vessel. In the dynamic mode, fresh solvent is continuously pumped at a selected flow rate for a fixed period of time. In both operating modes, the mass transfer rates are accelerated according to Fick’s law of diffusion [85].

PLE was found to be an alternative method to the conventional solvent extractions due to the short time of extraction, inert environment under high pressure and temperature [90]. Additionally, PLE is considered as a potential alternative technique to SFE for the extraction of polar compounds. Moreover, owing to the limited organic solvents used, PLE can be considered as a green extraction technique [91]. Therefore, PLE has commercial interest as alternative extraction method to obtain bioactive compounds from natural sources.

### 3.3. Anthocyanin Extraction and Separation Using Compressed Fluids

Compressed fluids-based extraction techniques, including subcritical and supercritical fluid approaches have been widely applied for the extraction of anthocyanins. Some of the most significant applications are listed in Table 2. Supercritical CO_2_ is a non-polar solvent, which limits its use in polar anthocyanin extraction. The addition of an organic co-solvents (ethanol, water and their mixtures) to supercritical CO_2_ is required to adjust the non-polar nature of supercritical CO_2_ and improve the affinity towards anthocyanins [86,92,93,94,95,96].

Ghafoor et al. investigated the effects of SFE temperature, pressure, and modifier concentration on the recovery of anthocyanins from grape (*Vitis labrusca* B.) peel [92]. The optimal SFE conditions were identified as 45 °C, 160 bar and 6–7% ethanol as modifier for a maximum total of anthocyanins of 1.2 mg/mL. These optimized conditions were comparable to those reported by Seabra et al. for the extraction of anthocyanins from elderberry pomace [97], and those reported by Paes et al. for blueberry residues [98], 40 °C and 200–210 bar using aqueous ethanol as a co-solvent. 

Another work involving the optimization of the extraction process parameters is given by Maran et al. The authors studied the influence of temperature, pressure and ethanol (co-solvent) flow rate on the supercritical CO_2_ extraction of anthocyanin from jamun (*Syzygium cumini* L.) fruits [96]. The highest total monomeric anthocyanin content of 231.3 ± 0.8 mg/100 g was achieved at 162 bar, 50 °C and 2 g/min of co-solvent flow rate. 

Jiao and Kermanshahi determined the total anthocyanin yield extracted by supercritical CO_2_ with water as co-solvent from haskap berry pulp at the optimized conditions of 450 bar, 65 °C, 15 min of static time, and 20 min of dynamic time to be 52.7% [93].

On the other hand, PLE has also been extensively applied for the recovery of anthocyanin from different natural matrices. Some of the most representative applications of this technique are shown in Table 2. In comparison with SFE, PLE covers a great range of compounds due to its flexibility in terms of solvents applied. For instance, Machado et al. studied the use of PLE in comparison with conventional methods to extract anthocyanins from blackberry (*Rubus fruticosus* L.) residues [99]. From the results, PLE exhibited a higher extraction rate than conventional techniques. The solvent type and temperature had strong influence on anthocyanins recovery. The higher anthocyanin yields (1.40 ± 0.02 and 1.39 ± 0.02 mg cyanidin-3-*O*-glucoside equivalent/g fresh residue) were achieved at 60 and 80 °C, respectively, and using pure ethanol or a mixture of ethanol and water (50% *v*/*v*) as solvents. 

Similar results were found for the extraction of anthocyanins from jabuticaba (*Myrciaria cauliflora*) skins [100] and from red grape (*V. labrusca* L.) pomace [101] using PLE. Extraction of jabuticaba skins with ethanol, at 50 bar and 80 °C, under a static extraction time of 9 min improved anthocyanins recovery (2.4 ± 0.5 mg cyanidin-3-*O*-glucoside/g dry material) as compared to the traditional method at reduced pressure. The extraction of anthocyanins from red grape pomace by PLE with mixture of ethanol and water (50% *v*/*v*) extracted more anthocyanins (497 ± 13 mg/100 g dry weight), working at an optimal PLE temperature of 100 °C. 

Another example of PLE application is given by Muangrat et al. who revealed that the total anthocyanin content of different dried parts of purple waxy corn (*Zea mays* L.) at the temperature of 100 °C and extraction time of 15 min increased with elevating the sample-to-solvent ratio (1:20–1:30) and water-to-ethanol ratio of (1:1–1:3). These extraction conditions yielded high total anthocyanin levels between 991 to 1552 µg cyanidin-3-*O*-glucoside/g dry weight of sample [102]. 

Another example of the influence of temperature on PLE is given by Saldaña et al. who demonstrated that extraction of anthocyanins from cranberry pomace was maximized using pressurized ethanol at 60–120 °C and 50 bar with a constant flow rate of 5 mL/min and extraction time of 10 min [101]. It was showed that there was no significant difference between anthocyanin content obtained after extraction of dried cranberry pomace from 40 to 120 °C at 50 bar. However, a significant decrease in anthocyanin content was observed at extraction temperatures from 140 to 160 °C, indicating that the ideal temperature range for anthocyanins extraction was 40–120 °C. This decrease in anthocyanin content is due to degradation of anthocyanins at higher temperatures (>140 °C) [20]. 

This behavior was also observed when anthocyanins were extracted from grape pomace, with the ideal extraction temperature ranging between 80–120 °C. Hence, it is clear that extraction temperature represents a key factor in the recovery of heat-sensitive anthocyanins.

Pressurized water at temperatures higher than the boiling point of water (100 °C) and lower than its critical temperature (374 °C) has also been applied to extract anthocyanins. Pressures between 35–200 bar are employed to maintain the water in the liquid state during the PLE process [91]. Since anthocyanins are thermo-labile compounds, the most important parameter affecting their recovery is the temperature and this should be taken into consideration when optimizing the process. Koyu et al. showed that for anthocyanins pressurized water extraction from *Morus nigra* L. fruits, the highest anthocyanin content (9.4 mg cyanidin-3-*O*-glucoside equivalent/g extract) was obtained at a low temperature (60 °C) [104].

Increasing the extraction temperature and decreasing water pH has been shown to improve the extraction of anthocyanins. Moreover, short-time extraction process is recommended to prevent possible degradation of anthocyanin moieties or their reaction with sugar and other products. In this sense, Ju et al. studied the extraction of anthocyanins from dried red grape pomace using subcritical water and subcritical sulfured water at different temperatures, ranging from 100 to 160 °C, for shorter periods of time (40 s). Higher levels of anthocyanins (59.3 ± 0.7 mg/g dry weight) were obtained at 110 °C compared to conventional solvent extraction at 60% methanol (50.2 ± 0.4 mg/g dry weight) [105]. 

Srinivas et al. investigated the influence of ethanol as entrainer, temperature, and organic acids on the subcritical water extraction of anthocyanins from sunbelt (*Vitis labrusca* L.) grape pomace. The pH 2.5 of the extraction solvent was maintained by using acetic, citric, formic, and tartaric acids. The anthocyanin yield was maximized (1028 mg/100 g DW) when using 80% (*v*/*v*) ethanol/water in presence of acetic acid at 80 °C [103].

Recently, the search for suitable clean solvents for PLE was extended. Loarce et al. improved considerably the recovery of anthocyanins from grape (*V. vinifera* L.) pomace using subcritical water extraction assisted by NADESs as modifiers. Different NADES, modifier percentage, and extraction temperature were screened. The highest anthocyanin content (11.2 ± 1.4 mg/L) was obtained with pressurized water assisted by 30% (*w*/*w*) of choline chloride and oxalic acid at 60 °C [106].

Some authors have compared the potential of subcritical liquids with supercritical CO_2_ approaches in the recovery of anthocyanins. As an example, Garcia-Mendoza et al. evaluated the extraction of anthocyanin from industrial residue of juçara (*Euterpe edulis* Mart.) using PLE and SFE. Anthocyanin-rich extracts (9.7 mg cyanidin-3-*O*-rutinoside equivalent/g dry residue) were obtained using PLE with acidified water at 40 °C. However, SFE using acidified mixture (pH 2) of ethanol and water (50% *v*/*v*) as co-solvent in a concentration of 10% (*w*/*w*), at 200 bar and 60 °C proved to be the most selective extraction process towards anthocyanins (22 mg cyanidin-3-*O*-rutinoside equivalent/g dry residue) [107]. Paes et al. also reported a significant improvement in the extraction of anthocyanins from blueberry residues by SFE compared with PLE [98]. However, different behavior was observed by other authors. Otero-Pareja et al. also investigated SFE and PLE to extract anthocyanins from wine industry wastes. The comparative study using different varieties of red grape pomace showed that PLE using hydro-alcoholic mixture (50% *v*/*v*) as solvent was more efficient than SFE using CO_2_ + 20% ethanol in terms of anthocyanins content, 742 ± 42 and 116 ± 2 mg malvin chloride/g dry extract, respectively [108]. 

Process intensification provides great opportunities to considerably improve the performance of extraction process. Sequential SFE/PLE or SFE integrated with PLE has been extensively studied as an efficient alternative to recover anthocyanins from a wide variety of natural sources. An example of this integrated process was given by Martinez et al. who reported a multiple step extraction process for anthocyanins from haskap berries with supercritical CO_2_ and ethanol as co-solvent. The developed process started with lipids separation with supercritical CO_2_ with 1 vol% ethanol, followed by terpenoids extraction with 10 vol% ethanol and finally the extraction of anthocyanins with >10 vol% ethanol. The sequential extraction yielded 7% more anthocyanins in comparison with conventional extraction [109]. Serra et al. applied a similar methodology to fractionate anthocyanins from cherries (*Prunus avium*), comprising a first step with supercritical CO_2_ followed by a second step where different mixtures of CO_2_ and ethanol [110]. 

Other approach of integrated process was developed by Seabra et al. and in this case, the authors investigated the extraction of anthocyanins from elderberry pomace by two-step extraction process using supercritical CO_2_ in a first step and mixture containing CO_2_, ethanol, and water in a second step [98]. The author observed that different proportion of CO_2_/ethanol/water had high impact on the extraction yield and composition of the extract in terms of total phenolic compound and anthocyanins. Similar multiple-step extraction strategies were performed to obtain anthocyanin-rich fractions from cranberry pomace [111] and bilberry (*Vaccinium myrtillus*) [112]. 

Another study of integrated SFE and PLE processes was followed by Monroy et al. A three-step extraction process using supercritical CO_2_, pressurized ethanol and pressurized water was applied to sequentially extract bioactive compounds from purple maize (*Zea mays* L.) pericarp. In this case, the pressurized aqueous fraction presented the highest anthocyanin content [113]. A similar approach was followed by Tamkute et al. who fractionated cranberry pomace [114]. 

An interestingly approach of integrated PLE process was performed by Pereira et al. that obtained an anthocyanin-rich fraction separately from other phenolic compounds present in grape (*Vitis vinifera* L.) marc using a sequential PLE process changing the solvent and increasing the temperature in the second step [115]. For anthocyanins, the best PLE condition was ethanol–water pH 2 (50% *w*/*w*) at 40 °C. For the recovery of phenolic compounds, the solvent was ethanol–water (50% *w*/*w*) at 100 °C. The first PLE step achieved 9.96 mg malvidin-3-*O*-glucoside/g dried grape marc. The mild temperature of this step prevented the anthocyanins thermal degradation before the second step, and low pH (pH 2) helped increasing their extraction yield.

## 4. Counter-Current Chromatography

**Figure 2:**
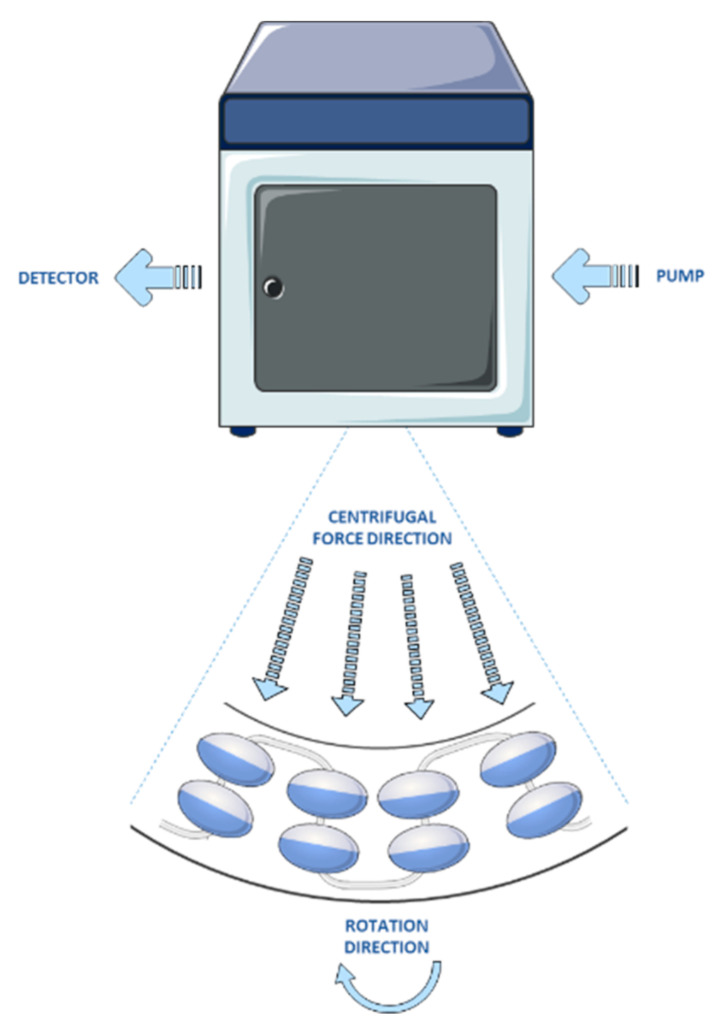
Schematic illustration of the counter-current chromatography process. This figure was made with some free images available on the Smart Servier Medical Art website (https://smart.servier.com, last accessed on 22 December 2021).

Counter-current chromatography is a support-free liquid–liquid chromatography based on the differential partitioning of compounds between two immiscible liquid phases of a solvent system. One of the phases is applied as a liquid stationary phase while the other as a mobile liquid phase [116]. During counter-current chromatography separation, the stationary phase is maintained in the column without the use of any solid support, whereas the immiscible mobile phase is pumped through the stationary phase. The counter-current chromatography column consists of a rotor containing series of tubes or chambers connected by ducts, where the two immiscible liquid phases are repeatedly mixed and settled (Figure 2) [117]. The different constituents of the sample are partitioned between two phases and eluted from the column at different time, according to their partition coefficient (K) [118].

In the modern counter-current chromatography techniques, introduced in the 1980s, centrifugal force fields are used to maintain the stationary phase inside the column. According to the different principles of fluid mechanics, modern counter-current chromatography systems can be classified into hydrodynamic, such as high-speed counter-current chromatography (HSCCC) [119,120], or hydrostatic, such as centrifugal partition chromatography (CPC) [121]. Hydrodynamic counter-current chromatography systems, pursued by Ito in the USA, are based on a variable centrifugal force field. The column contains one or several multilayer coils of open tubing mounted in a rotor that will induce a planetary rotation mode of the coils. The coils rotate around their axis which at the same time rotates around the central axis of the rotor [119]. Therefore, this technique is also known as multilayer coil countercurrent chromatography. In hydrostatic counter-current chromatography systems, pursued by Nunogaki in Japan, the column retains the liquid stationary phase in small chambers working with a constant centrifugal force field produced by a single axis rotation mechanism. The column comprise a series of channels connected in cascade by ducts and aligned in cartridges or disks in a rotor [122].

Comparing both counter-current chromatography systems, HSCCC columns are more efficient due to the number of theoretical plates, providing sharper peaks [123]. However, the retention of biphasic solvent systems with slight density difference between the phases could be low [116]. In contrast, CPC columns are usually more effective at retaining a broader range of biphasic solvents system, particularly the aqueous two-phase solvent system or some very hydrophilic systems. Moreover, the retention of stationary phase during the disruption of the equilibrium caused be high loadings and complex matrices is still high [123]. In addition, the CPC system allows working with faster mobile phase flow rates, reducing the separation time [116]. CPC apparatuses are easier to scale up without losing the chromatographic resolution. Nevertheless, CPC instrumentation is more expensive than HSCCC [122,124]. 

The liquid nature of the stationary phase presents several advantages when compared with the conventional chromatography techniques with solid stationary phase. In counter-current chromatography column, the stationary phase available for interaction with the solute can be higher than 80%, while in HPLC is only 20% [125], giving higher sample loading capacity and limiting the column overloading [116,126]. Moreover, the sample loss caused by irreversible adsorption associated with the solid matrix is avoided. Therefore, counter-current chromatography is characterized by high sample recovery without chemical modifications or loss of biological activity [126]. In addition, a multiple choice of biphasic solvent systems can be used in counter-current chromatography. This enables a large choice of solvents that can be employed with varying polarity that can ensure the proper dissolution of the target compounds. Thus, the crude extract can be loaded in the system without any clean-up pretreatment [127,128]. Moreover, the liquid stationary phase is more stable to acidic or basic conditions than silica-based supports [129]. Although counter-current chromatography efficiency is lower that HPLC, the high selectivity and the high stationary to mobile phase ratio largely compensate once both have a positive effect on resolution between the peaks [125]. From an economical point of view, counter-current chromatography has a relatively low cost of operating and maintenance by simplifying processing steps, decreasing solvent consumption (10 times less than for HPLC and dispensing expensive solid-support materials [122,128,130,131]. Once the initial investment in an instrument has been made, a set of coils would be expected to last the lifetime of the centrifuge. There are no column aging effects as a freshly-filled column of solvents is used in each experiment [125,128].

Despite many advantages, counter-current chromatography presents several challenges particularly associated with the retention of the liquid stationary phase in the column during the experiment. The stability of the stationary phase volume available to interact with the sample components is significantly affected by the counter-current chromatography column characteristics (column volume, tube/chamber number, size and shape), the biphasic solvent system physicochemical properties (density, viscosity, interfacial tension) and the operating conditions (choice of mobile and stationary phases, mobile phase flow rate, rotational speed and temperature) [132]. In fact, the counter-current chromatography separation efficiency and reproducibility are strongly dependent on the ability to retain the stationary phase in the chambers. Consequently, optimization of the biphasic solvent system and operating conditions is required for a successful counter-current chromatography separation.

Ito published a practical and effective guide for a step-by-step selection of counter-current chromatography optimum conditions comprising the selection of biphasic solvent systems, determination of K value, preparation of biphasic solvent system and sample solution, selection of elution mode, flow-rate, rotation speed, and on-line monitoring of the eluate [133].

The selection of a biphasic solvent system for a target compound is a critical step in the method development of a counter-current chromatography separation. Usually, the biphasic solvent system includes three or four solvents with different polarities [134]. The settling time of the biphasic solvent system should be shorter than 30 s to ensure a satisfactory retention of the stationary phase during the counter-current chromatography process. Compounds separation can be achieved by changing the ratio of the biphasic solvent system components to adjust their K value. The K value is the ratio of the target compound between both solvent phases. The counter-current chromatography “sweet spot” may be described as an area where K values are between 0.5 and 1.6 A compound with a smaller K value elutes closer to the solvent front with lower resolution while compounds with higher K values have higher resolution but longer elution time [135]. Moreover, the separation factor (α) between two compounds, i.e., the ratio of partition coefficients between two solutes, should be greater than 1.5 for successful separation [133]. Taking the above-mentioned considerations, several different and effective biphasic solvent systems have been proposed from least polar waterless systems to the most polar aqueous two-phase systems [128]. 

Once an appropriate biphasic solvent system is selected, the suitable operation mode for the separation has to be chosen. Both phases of the selected biphasic solvent system can be used as mobile phase, as long as the K value of the target compound is in the proper range. If the upper phase is used as mobile phase, counter-current chromatography is operating in the ascending mode. When the separation is accomplished with the lower phase as mobile phase, counter-current chromatography is working in the descending mode [124,136]. An exclusive feature of counter-current chromatography is the ability to switch between both modes, ascending and descending, during the experiment. The process that uses both modes to elute the solutes in the same separation is called dual-mode. When the procedure involves series of consecutive dual-mode steps, the method is named multi-dual mode elution. These methods can be applied when the target compounds present very different partition coefficient, accelerating the elution of compounds that have strong affinity with the stationary phase, reducing the separation time and improving the separation efficiency [124,127]. 

Counter-current chromatography displays greater flexibility in the choice of elution mode. The classical isocratic mode is a simple and effective method of isolating only few major compounds using a single biphasic solvent system. However, natural extracts typically contain a high number of different compounds with a broad range of polarity, requiring long separation time [86,127,137]. A suitable way to isolate compounds which differ widely in polarity and shorten the separation time is to apply a gradient elution mode with possible flow gradient, step gradient, polar gradient and pH gradient. During the separation with gradient elution mode, the composition of the mobile phase is changed, while the composition of the stationary phase undergoes minimum changes [138]. Nevertheless, modification of the mobile-phase composition during the gradient separation may disturb the biphasic solvent system equilibrium and hence the retained volume of stationary phase [116,125]. Thus, isocratic mode displays great advantage in the stability of stationary phase [124]. Besides the classical elution modes, other approaches based on displacement methods, such as strong ion-exchange and pH-zone refining, can be used. In this displacement mode, a retaining agent or ion exchanger is added to the stationary phase while a displacer is added to the mobile phase [127]. 

Finally, the flow rate of the mobile phase and rotation speed are process parameters with high influence on counter-current chromatography separation. The flow rate influences the separation time, the volume of stationary phase retained in the column, and consequently the peak resolution. Normally, lower flow rate provides higher retention of the stationary phase, improving the peak resolution while it requires a longer separation time. The use of a lower rotation speed will decrease the stationary phase volume retained in the column leading to lower peak resolution. On the other hand, the higher rotation speeds may produce excessive sample band due to the elevated pressure inside the column [133]. 

### Anthocyanin Separation Using Counter-Current Chromatography

Counter-current chromatography has been explored as an alternative to conventional techniques for fractionation of various classes of phenolic compounds including anthocyanins. The main application of counter-current chromatography includes separation and purification of anthocyanins from natural sources in large amounts required for compound identification, for further use as standards in analytical methods and for biological activity studies. Selected applications of counter-current chromatography applied to anthocyanins are shown in Table 3. 

HSCCC is by far the most used technique for anthocyanin separation and purification, followed by CPC. The most frequently used solvent system for the isolation of anthocyanin by HSCCC is tert-butyl-methyl ether, n-butanol, acetonitrile, and water acidified with TFA while ethyl acetate, n-butanol and water acidified with TFA is the most applied for CPC. Different modes of operation can be applied to anthocyanin fractionation including linear elution [139], gradient elution [140,141,142], pH zone refining, and strong ion exchange [143]. 

Renault et al. [140] presented, for the first time, the CPC application for the preparative purification of anthocyanins. The authors showed the isolation of malvidin-3-*O*-glucoside and peonidin-3-*O*-glucoside from Champagne vintage by-products (*Vitis vinifera*) by CPC in a single step. The biphasic solvent system ethyl acetate, n-butanol, and water were selected, taking into account the glycosidic structures of anthocyanins. Considering the complex composition of the extract, the separation was performed using a gradient elution in ascending mode, where the polar water rich phase was applied as the stationary phase, and the polarity of the organic mobile phase was varied by changing the ratio of ethyl acetate and n-butanol. In this way, the gradient elution was performed with the upper phase of ethyl acetate, n-butanol and water in a volume ratio of 77:15:8 and then, the upper phase of ethyl acetate, n-butanol and water in a volume ratio of 40:46:14. The stationary phase was the lower phase of ethyl acetate, n-butanol and water in a volume ratio of 5:5:90. All the phases were acidified with 0.8% of trifluoroacetic acid, in order to preserve the anthocyanins stability. Different fractions with different anthocyanin compositions were obtain. Fraction 1 containing 15 mg of pure peonidin-3-*O*-glucoside, fraction 2 presenting 20 mg of pure malvidin-3-*O*-glucoside. The intermediate fractions (2 mg) containing a mixture of these two compounds. This strategy was successfully scaled-up to a multi-gram scale on a 5 L pilot CPC chromatograph by Renault et al. [141]. Moreover, the authors generalized the use of the described gradient elution with the same biphasic solvent system ethyl acetate, n-butanol and water for separation of anthocyanins from blackcurrant (Ribes nigrum L.). Additionally, two other studies reported the application of this gradient elution method for the fractionation of anthocyanins from Calafate Berries (*Berberis microphylla* G. Forst) [144] and grapes (*Vitis vinifera* L.) [145] with anthocyanins being fractionated and purified using a combination of CPC and preparative HPLC. 

CPC was also successfully applied to the isolation of delphinidin-3-*O*-sambubioside-5-*O*-glucoside from a crude extract of Maqui Berry (*Aristotelia chilensis*), as reported by Rojo et al. [139]. The solvent system used for the separation consisted of ethyl acetate, n-butanol and water in a volume ratio of 2:3:5 acidified with 0.1% of trifluoroacetic acid. Pure delphinidin-3-*O*-sambubioside-5-*O*-glucoside (6.76% of the anthocyanin extract) was obtained combining CPC with preparative HPLC. 

Yousof et al. [143] reported the use of CPC to the isolation of anthocyanins from *Hibiscus sabdariffa* L. using the biphasic solvent system ethyl acetate, n-butanol and water acidified with trifluoroacetic acid (organic mobile phase volume ratio of 4.0:4.6:1.4 and aqueous stationary phase volume ratio of 0.5:0.5:9.0). The authors compared the standard elution with two displacement modes: strong ion-exchange and pH-zone refining. Among the three tested strategies, strong ion-exchange using trioctylmethylammonium chloride as an exchanger was the most effective in purifying Hibiscus sabdariffa L anthocyanins. From 1 g of crude extract, 80 mg of delphinidin-3-*O*-sambubioside, 40 mg of cyanidin-3-*O*-sambubioside, 15 mg of delphinidin-3-*O*-glucoside and 4 mg of cyanidin-3-*O*-glucoside were obtained after 75 min.

Recently, the search for suitable clean solvents for counter-current chromatography processes has been expanded. Ionic liquids and deep eutectic solvents are some examples of solvents reported as environmentally acceptable options for various aspects of chromatographic techniques, including stationary phase and mobile phase [145]. In this line, Lima et al. investigated the use of an aqueous two-phase system based on protic ionic liquid (bis(2-hydroxyethyl)ammonium hydrogen sulfate) and acetonitrile to fractionate anthocyanins from grape pomace. The best condition corresponded to a purification factor of 41.88-fold [146]. This study demonstrated the CPC compatibility with new alternative green solvents systems.

## 5. Conclusions

Recent trends in the extraction techniques have mainly been focused on developing cost-effective and eco-friendly extraction methodologies for selective recovery of targeted compounds at lesser extraction time from renewable sources. Anthocyanins are mainly extracted trough conventional methods that are time-consuming and involve the use of organic solvents. Moreover, the chemical diversity of the obtained complex extracts make the downstream purification step challenging. In this review, the most significant development on the advanced techniques for anthocyanins extraction, such as SFE, PLE, and DES, have been summarized. Furthermore, counter-current chromatography has been presented as an alternative downstream process for anthocyanin purification from crude extracts.

SFE using food-grade organic modifiers is a proven sustainable and environmentally friendly technique that limits the use of toxic organic solvents, improves the selectivity towards anthocyanins adjusting the fluid density, and preserves anthocyanins bioactivity due to the absence of oxygen in the system and low operating temperatures. PLE has been gaining popularity as a green method for anthocyanins extraction due to its automation, which allows high extraction yields in shorter time with minimum solvent consumption. Short-time extraction processes are preferred in order to prevent possible degradation of anthocyanins. Besides, SFE and PLE are scalable and can be integrated to other processes within a biorefinery or intensification approach. 

DES have become an interestingly alternative solvents for anthocyanin recovery since they can be tunable for a particular purpose. In addition, the extraction procedure using DES is very simple and specialized equipment is not required. 

However, these alternative extraction technologies present some challenges that need to be addressed. These difficulties include the high susceptibility of matrix effects that requires optimization of extraction process in each case, the extraction of highly polar anthocyanin using SFE, thermal degradation of the anthocyanins when using of PLE, the interference of DES viscosity on anthocyanin extraction efficiency, the high cost of high-pressure equipment and qualified manpower to operate the machinery required for both compressed fluid-based extraction techniques, and challenges in the industrial scale application of pressurized hot water extraction.

Counter-current chromatography is a developing technique for downstream isolation of anthocyanins from complex mixtures that presents great efficiency and high recovery ability. The separation can be carried out in acidic media and in the absence of oxygen, preserving anthocyanins bioactivity. However, some of the organic solvents that are commonly used in CCC are toxic and their use is not allowed in food, nutraceuticals, and cosmetic formulations. Moreover, the high cost of counter-current chromatography equipment can be a challenge in the industrial application of this technique. With the increasing popularity of counter-current chromatography for the bioactive compounds purification, developing of greener biphasic solvent systems for anthocyanins separation is necessary. Since DESs have been reported as promising alternatives to traditional volatile solvents, their potential for use in counter-current chromatography processes as stationary and mobile phase is worth exploring. In this way, integrating counter-current chromatography with NADESs can reduce the use of organic solvents and thus making this technique more economically and environmentally feasible. Therefore, an interesting trend in the application of the revised techniques is the development of integrated processes. When integrated, these technologies can offer a powerful approach for the separation of anthocyanins.

## Figures and Tables

**Table 1:** Some examples of DES used for the extraction of anthocyanins and respective extraction yields (ChCl-choline chlorine; LA-lactic acid; Oa-Oxalic acid; Pro-Propylene glycol; TA-tartaric acid; CA-citric acid; MA-malic acid; gly-glycerol; Eg-Ethylene glycol).

Source of Anthocyanins	DES			Preparation and Auxiliary Extraction Techniques	Anthocyanin Content (mg/g)	Ref.
	Composition	Molar Ratio	Water (%)			
Frozen blueberry peels	ChCl:LA	1:1	22	Stirring and heating at 50 °C for 2 h (Microwave and ultrasound-assisted)	23.59	[51]
Grape skin	ChCl:Oa	1:1	25	Stirring and heating at 80 °C for 2–6 h (Ultrasonic processor-assisted)	~12	[52]
*Hibiscus sabdariffa* L. calyces	ChCl:Oa	1:1	25	Heating at 80 °C (Microwave-assisted)	3.76 ± 0.03 (delphinidin-3-sambubioside) 3.60 ± 0.03 (cyanidin-3-sambubioside)	[46]
Cranberry pomace	Glucose:LA	1:5	20	Stirring and heating at 50 °C for 30 min (Ultrasonic processor-assisted)	1.54	[53]
Jabuticaba pomace	ChCl:Pro	1:2	-	Heating at 80 °C for 21 min	~2.8 (expressed as Monomeric Anthocyanin Pigment)	[45]
Mulberry fruits	ChCl:LA	1:2	3.19	Stirring and heating at ≤100 °C followed by drying in a vacuum oven at 45 °C	4.24 ± 0.20	[54]
Blue honey-suckle fruits	ChCl:LA	1:2	20	Stirring and heating at 80 °C	5	[55]
Red wine by products	ChCl:TA	1:2	44	Stirring and heating at 90 °C	3.33	[56]
Black carrots	ChCl:CA	1:1	-	Stirring and heating at 80 °C for 2–6 h (Ultrasound-assisted)	6	[57]
*L. chequen* (Molina) A. Gray	Glu:LA	1:8	-	Storage at 80 °C for 2 h followed by lyophilization for 18–24 h until a homogeneous and viscous liquid was obtained. (Ultrasound-assisted)	3.30	[58]
Wine lees	ChCl:MA	-	34.5	Stirring and heating at 80 °C for 120–360 min (Ultrasound-assisted)	6.55	[47]
Mulberry fruits	ChCl:CA	1:1	30	Stirring and heating at 80 °C (High-speed homogenization and cavitation-burst assisted)	~5.50	[59]
Blackcurrant	ChCl:LA	1:2	20	Stirring and heating at 80 °C (Microwave assisted)	~2.0	[60]
Blueberry	ChCl:gly:CA	0.5:2:0.5	25	Stirring and heating at 80 °C for 30 min	2.30	[61]
Blueberry pomace	ChCl:Oa	1:1	30	Stirring and heating at 80 °C (pulse-ultrasonication assisted extraction)	~25	[62]
*Ixora javanica* flower	ChCl:Eg	1:2	-	Stirring and heating at 50 °C for 30 min (Ultrasound-assisted)	~13	[63]
*Raspberry*	ChCl:1,4-butanediol	1:3	29	Stirring and heating at 80 °C (Ultrasound-assisted)	1.378 ± 0.009	[64]

**Table 2:** Selected studies on separation of anthocyanins using compressed fluids.

**Source of Anthocyanin**	**Technique**	Solvent	Modifier	Temperature (°C)	Pressure (bar)	Flow Rate	Time	Anthocyanin Content/Yield	Ref.
Grape (*Vitis labrusca* B.) peel	SFE	CO_2_	6–7% Ethanol	45–46	160–165	2 mL/min	30 min	1.2 mg/mL	[92]
Indian blackberry (*Syzygium cumini* L.)	SFE	CO_2_	Ethanol	50	162	2 g/min	n.d.	231.3 ± 0.8 mg/100 g	[96]
Haskap berry pulp	SFE	CO_2_	5.4 g Water/3.2 g berry pulp paste	65	450	10 mL/min	15 min static, 20 min dynamic	52.7% anthocyanins yield	[93]
Blackberry residues	LE	Ethanol	-	60	75	3.80 mL/min	30 min	1.40 ± 0.02 mg cyanidin-3-*O*-glucoside equivalent/g fresh residue	[99]
	PLE	Ethanol/water (50% *v*/*v*)	-	80	75	3.35 mL/min	30 min	1.39 ± 0.02 mg cyanidin-3-*O*-glucoside equivalent/g fresh residue	
Jabuticaba skins	PLE	Ethanol	-	80	50	Static	9 min	2.4 ± 0.5 mg cyanidin-3-*O*-glucoside/g dry material	[100]
Red Grape Pomace	PLE	Ethanol (50–70% *v*/*v*)	-	100	68	Static	n.d.	497 ± 13 mg/100 g dry weight	[101]
Purple waxy corn (*Zea mays* L.)	PLE	Ethanol (50–75% *v*/*v*)	-	100	n.d.	Static	15 min	991 to 1552 µg cyanidin-*O*-3-glucoside/g dry weight	[102]
Cranberry pomace	PLE	Ethanol	-	40	50	5 mL/min	10 min	6.3–7.8 mg cyanidin-3-*O*-glucoside equivalent	[20]
Grape pomace	PLE	Ethanol/water (80% *v*/*v*)	-	80	103	Static	1 min	1028 mg/100 g dry weight	[103]
*Morus nigra* L. fruits	PLE	Water	-	60	150	2 mL/min	60 min	9.4 mg cyanidin-3-*O*-glucoside equivalent/g extract	[104]
Red grape skin	PLE	Water	Sodium metabisulfite (1400 µg/mL)	100	n.d.	Static	40 s	59.3 ± 0.7 mg/g dry weight	[105]
Grape pomace	PLE	Water	Choline chloride and oxalic acid (30% *w*/*w*)	60	n.d.	Static	2 cycles of 10 min	11.2 ± 1.4 mg/L	[106]
SFE vs. PLE									
Juçara residues	SFE	CO_2_	10% Ethanol/water (50% *v*/*v*, pH 2)	60	200	2.08 × 10^−4^ kg/s	7 min static, 39 min dynamic	22 mg cyanidin-3-*O*-rutinoside equivalent/g dry residue	[107]
	PLE	Water (pH 2)	-	40	100	1.5 mL/min	n.d.	9.7 mg cyanidin-3-*O*-rutinoside equivalent/g dry residue	
Blueberry residues	SFE	CO_2_	10% Ethanol/water (50% *v*/*v*)	40	200	10 mL/min	n.d.	1071 ± 64 mg/100 g	[98]
	PLE	Ethanol/water (50% *v*/*v*, pH 2)	-	40	200	10 mL/min	n.d.	254.0 ± 0.6 mg/100 g	
Red Grape Pomace	SFE	CO_2_	20% Ethanol	55	100	25 g/min	3 h	116 ± 2 mg malvin chloride/g dry extract	[108]
	PLE	Ethanol/water (50% *v*/*v*)	-	120	100	5 g/min	3 h	742 ± 42 mg malvin chloride/g dry extract	
**Integrated process**									
Haskap berries	1st step: SFE	CO_2_	1% Ethanol	50	300	2 mL/min	30 min	Lipophilic fraction	[109]
	2nd step: SFE	CO_2_	20% Ethanol	50	300	2 mL/min	1 h static, 1 h dynamic	Terpenoids fraction	
	3rd step: SFE	CO_2_	50% Ethanol	50	300	2 mL/min	1 h static, 1 h dynamic	Anthocyanins fraction	
Sweet cherry (*Prunus avium*)	1st step: SFE	CO_2_	-	50	250	n.d.	15 min static, 1 h dynamic	Lipophilic fraction	[110]
	2nd step: PLE	100% Ethanol	-	50	250	n.d.	1.5 h	0.99 ± 0.05 mg cyanidin-3-*O*-glucoside/g	
Elderberry pomace	1st step: SFE	CO_2_		40	210	12.3 ± 1.4 × 10^−5^ kg/s	15 min static, 40 min dynamic	Lipophilic fraction	[97]
	2nd step: SFE	CO_2_	CO_2_/ethanol/water (80:1:19) and (60:8:32)	40	210	7.2 ± 0.4 × 10^−5^ kg/s	45 min dynamic	12.0–13.3% total anthocyanins	
Bilberry (*Vaccinium myrtillus*)	1st step: SFE	CO_2_	6% Ethanol/water (70% *v*/*v*)	45	250	8 kg/h	1 h		[111]
	2nd step: SFE	CO_2_	6% Ethanol/water (50% *v*/*v*)	45	250	6 kg/h	1 h		
	3rd step: SFE		9% Ethanol/water (10% *v*/*v*)	45	250	6 kg/h	3 h	0.62 ± 0.05 mg/g dw anthocyanins	
Cranberry pomace	1st step: SFE	CO_2_	-	50	400	1 L/min	180 min	Lipophilic fraction	[112]
	2nd step: SFE	CO_2_	CO_2_/ethanol/water (0.312:0.048:0.640)	50	400	1 L/min	420 min	84.6 ± 2.3% Total anthocyanin	
Purple corn (*Zea mays* L.)	1st step: SFE	CO_2_	-	50	400	1.6 g/min	90–100 min	Lipophilic fraction	[113]
	2nd step: PLE	Ethanol	-	50	400	0.5 mL/min	150–220 min	63.8–63.1 mg/g total monomeric anthocyanins	
	3rd step: PLE			50	400	0.5 mL/min	150–220 min	41.3–54.6 mg/g total monomeric anthocyanins	
Cranberry pomace	1st step: SFE	CO_2_	-	53	424	n.d.	158 min	Lipophilic fraction	[114]
	2nd step: PLE	Ethanol	-	70	103	Static	3 cycles, 10 min	9.0 ± 1.1 mg/g extract 416 ± 51 mg/100 g dry weight	
Grap marc	1st step: PLE	Ethanol/water (50% *w*/*w*, pH 2)	-	40	100	5 g/min	40 min	10.0 mg malvidin-3-*O*-glucoside/g of dried grape marc	[115]
	2nd step: PLE	Ethanol/water (50% *w*/*w*)	-	100	100	5 g/min	40 min	Phenolic fraction	

**Table 3:** Selected studies on separation of anthocyanins using counter-current chromatography (EtOAc: ethyl acetate; BuOH: n-butanol; W: water; TFA: trifluoroacetic acid; ACN: acetonitrile; [2HEA]HSO_4_]: bis(2-hydroxyethyl)ammonium hydrogen sulfate).

Source of Anthocyanin	Solvent System	Column Capacity (mL)	Flow Rate (mL/min)	Rotation Speed (rpm)	Ref.
Champagne vintage by-products	EtOAc:BuOH:W, 0.8% TFA	240	3	1300–1500	[140]
	Stationary phase—5:5:90				
	Initial mobile phase—77:15:8				
	Final mobile phase—40:46:14				
	Ascending				
Champagne vintage by-products	EtOAc:BuOH:W, 0.2% TFA	5470	60	1140	[141]
	Stationary phase—5:5:90				
	Initial mobile phase—77:15:8				
	Final mobile phase—40:46:14				
	Ascending				
Blackcurrant (*Ribes nigrum* L.)	EtOAc:BuOH:W, 0.2% TFA	230	3	1400	[141]
	Stationary phase—5:5:90				
	Initial mobile phase—77:15:8				
	Final mobile phase—40:46:14				
	Ascending				
Calafate berry (*Berberis microphylla* G. Forst)	EtOAc:BuOH:W, 0.1% TFA	200	5	n.d.	[144]
	Stationary phase—4:5:91				
	Initial mobile phase—77:15:8				
	Final mobile phase—40:46:14				
	Ascending				
Grape skin (*Vitis vinifera* L.)	EtOAc:BuOH:W, 0.1% TFA	200	3	1000	[145]
	Stationary phase—5:5:90				
	Initial mobile phase—77:15:8				
	Final mobile phase—40:46:14				
	Ascending				
Grape skin (*Vitis vinifera* L.)	EtOAc:BuOH:W, 0.1% TFA	1000	15	1000	[145]
	Stationary phase—5:5:90				
	Initial mobile phase—77:15:8				
	Final mobile phase—40:46:14				
	Ascending				
Maqui Berry (*Aristotelia chilensis*)	EtOAc:BuOH:W, 0.1% TFA	1000	n.d.	n.d.	[139]
	2:3:5				
	Ascending				
*Hibiscus sabdariffa* L.	EtOAc:BuOH:W, 0.5% TFA	200	10	1500	[143]
	Stationary phase—5:5:90				
	Initial mobile phase—77:15:8				
	Final mobile phase—40:46:14				
	Ascending				
*Hibiscus sabdariffa* L.	EtOAc:BuOH:W	200	10	1500	[143]
	Stationary phase—5:5:90, 20 mM NaOH (pH~10)				
	Initial mobile phase—77:15:8, 16 mM TFA (pH~2)				
	Final mobile phase—40:46:14, 16 mM TFA (pH~2)				
	Ascending, pH-zone refining				
*Hibiscus sabdariffa* L.	EtOAc:BuOH:W	200	10	1500	[143]
	Stationary phase—5:5:90, quaternary ammonium salt (Aliquat 336™)				
	Initial mobile phase—77:15:8, NaI				
	Final mobile phase—40:46:14, NaI				
	Ascending, strong-on exchange				
Grape pomace	ACN:[2HEA]HSO4]:W	50	1.5	2500	[146]
	5:1:4				
	Ascending				
